# Increased leukotoxin production: Characterization of 100 base pairs within the 530 base pair leukotoxin promoter region of *Aggregatibacter actinomycetemcomitans*

**DOI:** 10.1038/s41598-017-01692-6

**Published:** 2017-05-15

**Authors:** Vandana Sampathkumar, Senthil Kumar Velusamy, Dipti Godboley, Daniel H. Fine

**Affiliations:** Department of Oral Biology, Rutgers School of Dental Medicine, Newark, NJ 07103 USA

**Keywords:** Bacterial genes, Pathogens

## Abstract

*Aggregatibacter actinomycetemcomitans* leukotoxin (LtxA) is a major virulence factor that kills leukocytes permitting it’s escape from host immune surveillance. *A*. *actinomycetemcomitans* strains can produce high or low levels of toxin. Genetic differences reside in the “so called JP2” *ltxA* promoter region. These hyper-leukotoxin producing strains with the 530 bp deletion have been studied in detail. However, regions contained within the 530 bp deletion that could be responsible for modulation of leukotoxin production have not been defined. Here, we report, for the first time, on regions within the 530 bp that are responsible for high-levels of *ltx*A expression. We constructed a deletion of 530 bps in a primate isolate of *A*. *actinomycetemcomitans*, which produced leukotoxin equivalent to the JP2 strain. We then constructed sequential deletions in regions that span the 530 bps. Results indicated that expression of the *ltxA* transcript was reduced by a potential transcriptional terminator in promoter region 298 to 397 with a ΔG = −7.9 kcal/mol. We also confirmed previous findings that transcriptional fusion between the *orf*X region and *ltx*C increased *ltx*A expression. In conclusion, we constructed a hyper-leukotoxin producing *A*. *actinomycetemcomitans* strain and identified a terminator located in the promoter region extending from 298–397 that alters *ltx*A expression.

## Introduction

Leukotoxin (LtxA), is considered as one of the major virulence factors produced by *Aggregatibacter actinomycetemcomitans*. This large pore forming protein helps *A*. *actinomycetemcomitans* evade the host immune system by killing neutrophils, lymphocytes, and monocytes^1, 2^ and thus protects *A*. *actinomycetemcomitans* against surveillance and destruction by its native host^3^. Two major strains of *A*. *actinomycetemcomitans* have been reported, a minimal leukotoxin producing strain (652 type) and hyper-producing leukotoxin strain (JP2 type)^4^. At the genetic level the hyper-producing strain shows a deletion of 530 bp in the promoter region that appears to be responsible for increased expression of downstream *ltx* genes^4^. Regulation of *ltx*A expression has been studied extensively at the molecular level in naturally occurring promoter-deleted strains described as JP2-like strains^4–12^. Studies have shown that this deletion appears to, 1) upregulate *ltx*A expression, and 2) result in differential transcription that can influence *ltx*A expression. These studies have been performed, for the most part, in JP2-like strains that; 1) lack many characteristics seen in recently isolated clinical isolates, and 2) are devoid of the 530 bp promoter region thought to be critical for elevated leukotoxin production^8^. Comparison between a minimal leukotoxin producing wild-type (WT) strain, and a hyper-producing strain derived from the same WT parental strain have not been studied. It was our belief that the sequential analysis of deletions within the missing 530 bp region and the relationship of these missing regions with regulation of *ltx*A could be assessed in these genetically re-constructed strains. Taken together, it would seem likely that comparison between a WT parental strain containing the full-length promoter region and strains with a series of deletions in the promoter region derived from its parent strain should indicate how specific regions within the 530 bp could influence leukotoxin expression.

Over the last 30 years there have been a number of studies designed to understand the role of the promoter region in leukotoxin production^12^. Virtually all of the work has been described using the JP2-like strain as compared to an unrelated 652 minimal leukotoxin producing strain containing the wild-type promoter^4, 8, 13, 14^. Hence, most comparisons are based on speculation and abductive inference^15^. However, since these studies were conducted in the JP2-like strain they focused on the role of regulatory proteins that might bind to DNA upstream from the missing promoter region^5, 6, 10^. Regulatory proteins such as CRP, (cAMP Receptor Protein)^9^, IHF (Integration Host Factor)^12^ were recognized as either positive and/or negative regulators of *ltx*A expression respectively. Further, Mlc was identified as an activator of *ltx*A^11^.

Another proposal suggested that the 530 bp deletion shortens the interval between an RNA polymerase binding site and *ltx*A structural genes, which implies that increased transcription could occur as a result of this shortened distance^15^. Environmental conditions have also been shown to influence leukotoxin production. As such, increased cAMP levels, lower pH (6.0–7.0) and growth under anaerobic conditions have all been shown to increase leukotoxin transcription^8, 16–18^. Since the periodontal pocket is more anaerobic at its deepest point these suggestions appear to be relevant. Moreover, the oxygen tension in the pocket is known to decrease during infection in the sub-gingival region. Nevertheless, even though regulatory proteins have been identified, there are still a significant number of unanswered questions relating to *ltx*A expression. Chief among them is definitive proof that a specific portion of the deleted DNA promoter region affects leukotoxin expression levels^4, 8–10, 12, 17^.

The strains used in this study were derived from a Rhesus (Rh) monkey. Our long-term study goal was to assess a number of *A*. *actinomycetemcomitans* virulence genes and to determine their effect on *A*. *actinomycetemcomitans* colonization in the mouths of Rh monkeys. As such we deleted *lux*S and *ltx*A and studied these deletions *in vivo* and *in vitro*. The *in vitro* results presented herein indicate that the entire 530 bp deletion is not mandatory for excessive LtxA production. Furthermore, we found that a key determinant for expression of leukotoxin is found in a 100 bp sequence in the promoter region that contains a terminator, which when deleted permits high levels of production.

## Results

### Construction of a hyper LtxA producing *A. actinomycetemcomitans* from a minimal leukotoxin producer

**Figure 1:**
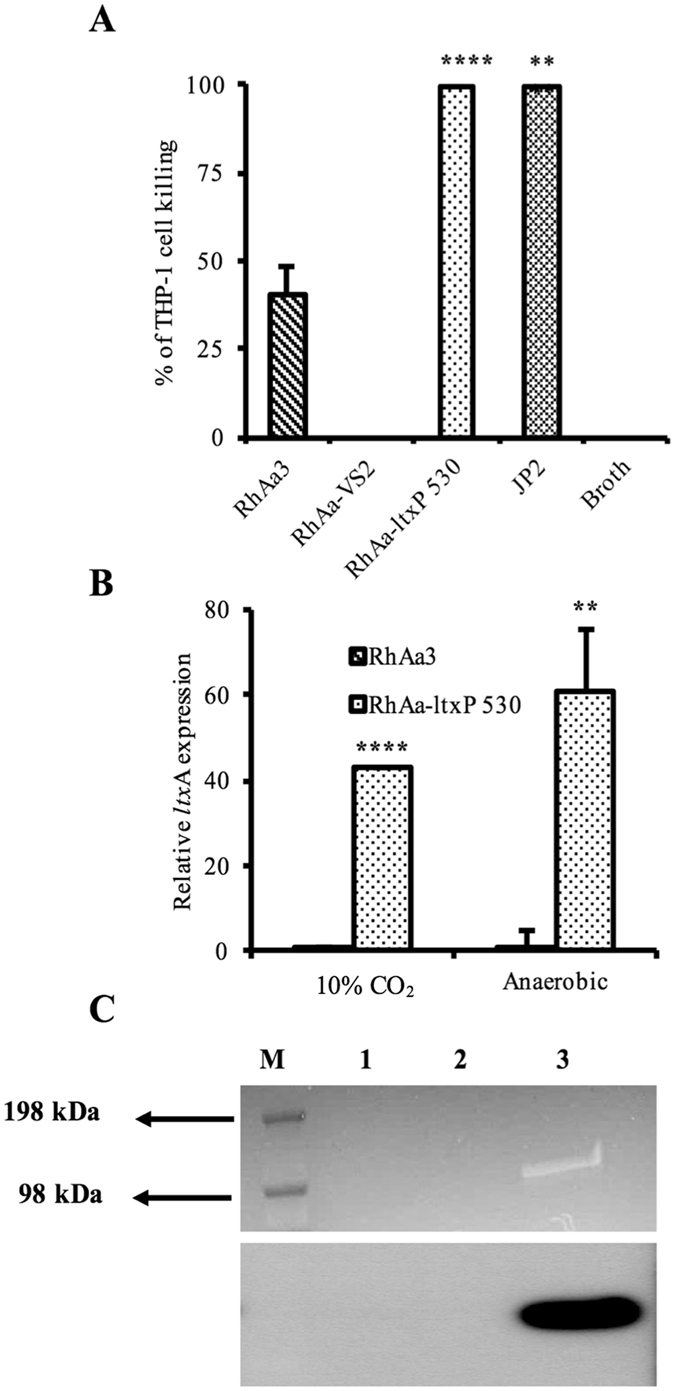
Leukotoxin expression and activity of constructed RhAa-*ltx*P530 strain. The leukotoxic activity of wild type RhAa3 and hyperproducer, RhAa-*ltx*P530 were tested in extracellular extracts against human THP-1 macrophages. The RhAa-*ltx*P530 strain showed significantly higher leukotoxic activity than extract from RhAa3. The JP2 strain extracellular extract used as the positive control, and uncultured growth media was used as the negative control. The significant difference in the leukotoxic activity (*P < 0.05 ± SD) between *A*. *actinomycetemcomitans* strains were calculated by One-way ANOVA test with Tukey’s multiple comparison post-hoc test. All experiments were conducted in biological triplicates (**A**). qRT-PCR analysis of *ltx*A expression in anaerobic and. The significant fold change (*P < 0.05) between RhAa3 and RhAa-*ltx*P530 was calculated by Student’s *t*-test (**B**). SYPRO^®^ ruby staining (Top panel) and western blot (bottom panel) showing RhAa-*ltx*P530 produced high levels of leukotoxin as compared to RhAa3. The RhAa-VS2, a *ltx*A disrupted mutant was used as the negative control. Since the RhAa3 wild type produces minimal leukotoxin it cannot be seen in western blot (M-Marker; 1-RhAa3; 2-RhAa-VS2; 3-RhAa-*ltx*P530 (**C**).

Our principal goal is to study the role of different virulence factors of *A*. *actinomycetemcomitans in vivo* in a real world Rh monkey model. In this context, a previous study showed that a LtxA null producer failed to colonize the oral cavity of Rh monkeys whereas the wild-type strain RhAa3 colonized^19^. The initial aim of the current study was to develop a hyper LtxA producing strain from the same wild-type parental strain for testing in our monkey model. The hyper LtxA producing RhAa-*ltx*P530 strain was developed as described in the methods section. To assess the LtxA levels, RhAa-*ltx*P530 biofilm cells were grown alongside RhAa3 (wild type strain), while the RhAa-VS2 (a leukotoxin mutant strain) was used as a negative control. Both the JP2 strain and the RhAa-*ltx*P530 (the test strain) exhibited significantly high levels of leukotoxin activity (*P* < 0.0001) as compared to the RhAa3 strain (Fig. 1A). Further, qRT-PCR analysis of *ltx*A showed that there was a significantly high level of expression (*P* < 0.0001) in RhAa-*ltx*P530 strain as compared to minimal LtxA production in wild type RhAa3 (Fig. 1B). We also analyzed leukotoxin levels in an anaerobic cell free supernatant by SDS-PAGE SYPRO^®^ ruby staining and western blot. Results showed an intense band in RhAa-*ltx*P530 strain corresponding to the molecular weight of ~113 kDa, which reacted with antibody to leukotoxin (Fig. 1C).

### Leukotoxin promoter region 298–397 is important in controlling leukotoxin expression

**Figure 2:**
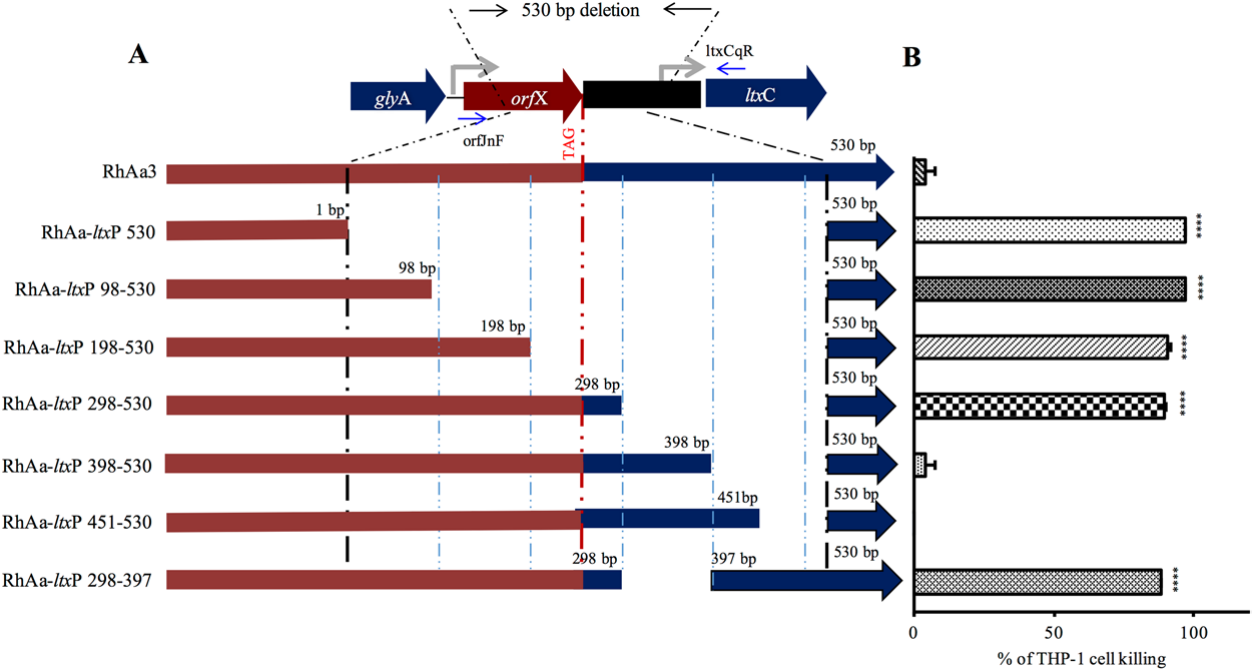
Physical map of the constructed *ltx*A promoter deletion strains and their leukotoxic activity. The physical map showing the region deleted within the 530 bp. 1 bp indicates the start of 530 bp deletion in the *orf*X and the respective number of base pairs deleted are indicated. 530 bp indicates the end of 530 bp deletion similar to a JP2 type strain. Bold lines indicate the region without deletion and thin line indicate the regions deleted. A red dotted vertical line represents the stop codon of *orf*X (**A**). The cell free culture supernatants were tested for the leukotoxic activity from 24 h biofilm growth of different strains. The RhAa-*ltx*P530, RhAa-*ltx*P98-530, RhAa-*ltx*P198-530, RhAa-*ltx*P298-530 and RhAa-*ltx*P298-397 showed significantly higher killing than the RhAa3 strain. Values indicate the mean percentages of human macrophage THP-1 cell killing performed in independent triplicate experiment. Error bars indicate standard deviation (SD). The significant killing of THP-1 cells between different *A*. *actinomycetemcomitans* strains were calculated by One-way ANOVA test. *P < 0.001 ± SD with (**B**).

A series of deletions were made in the leukotoxin promoter region. A physical map of mutations created in the *ltx*A promoter region as shown in Fig. 2A. To test the leukotoxic activity due to different mutations in the promoter region, a THP-1 functional cell killing assay was performed. The results indicated that strains RhAa-*ltx*P98-530 (*P* < 0.0001), RhAa-*ltx*P198-530 (*P* < 0.0001) and RhAa-*ltx*P298-530 (*P* < 0.0001) exhibited significantly increased leukotoxic activity when compared to RhAa3 (Fig. 2B). However, RhAa-*ltx*P398-530 and RhAa-*ltx*P451-530 did not show significantly increased leukotoxin activity when compared to RhAa3 (Fig. 2B) suggesting that the region between 298–397 could be critical in controlling leukotoxin expression. Therefore, another deletion was carried out in which the region 298–397 was removed and the resultant strain RhAa-*ltx*P298-397 was assessed for leukotoxin activity. Interestingly, the strain RhAa-*ltx*P298-397 produced significantly high leukotoxic activity (*P* < 0.0001) compared to RhAa3 strain (Fig. 2B). Since the RhAa-*ltx*P298-397 strain showed significantly increased leukotoxic activity, it was proposed that this region has the potential site for a negative regulatory element binding that could alter leukotoxin expression.

### Analysis of the mutant strains for the transcriptional fusion in *orf*X-*ltx* operon

**Figure 3:**
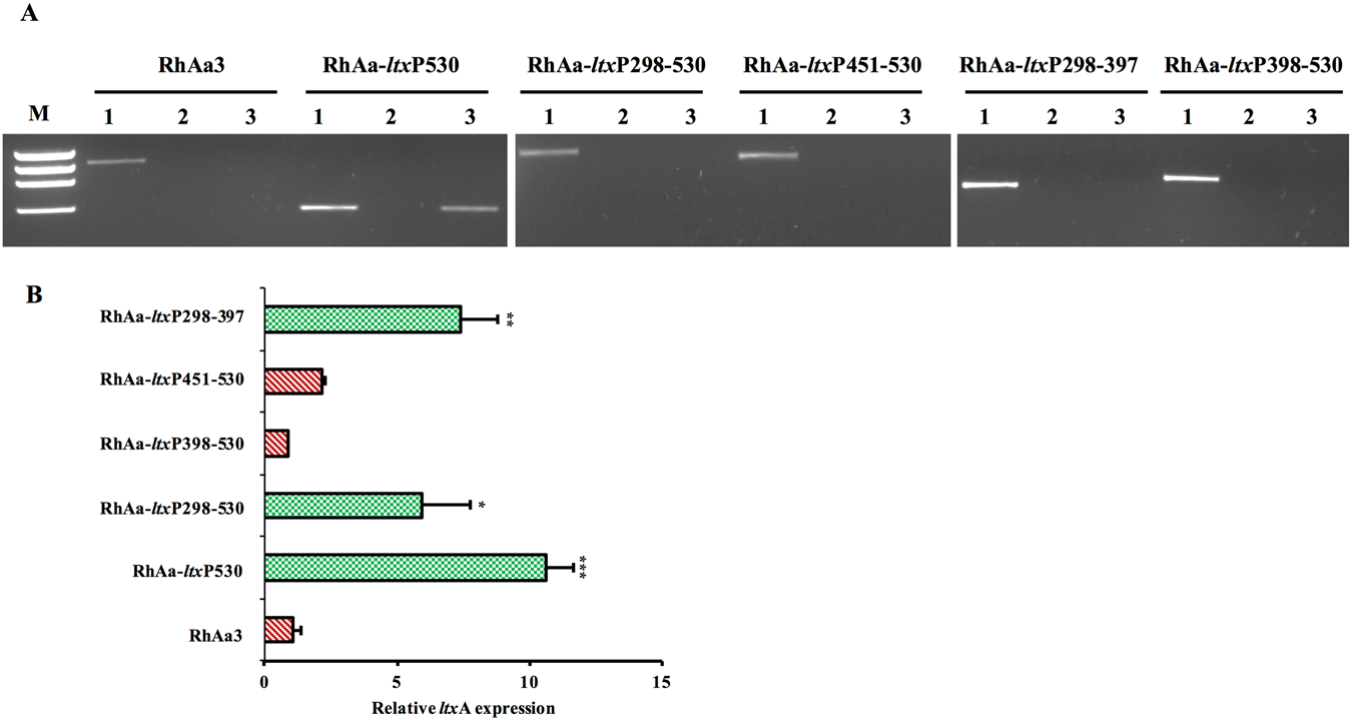
Transcriptional fusion of *orf*X with *ltx* operon due to promoter region deletion. A representative RT-PCR gel picture showing the transcriptional fusion in RhAa-*ltx*P530, but not in RhAa3 and RhAa-*ltx*P298-397 is seen. 1 – Genomic DNA template (+ve control); 2 – RNA template with no RT (−ve control); 3 – cDNA template (**A**). qRT-PCR analysis was carried out to assess the expression level of *ltx*A due to promoter deletion. There was a 10.7-fold increase in *ltx*A expression in RhAa-*ltx*P530, 5.9 fold increase in RhAa-*ltx*P298-530 and 7.4 fold increase in RhAa-*ltx*P298-397 compared to RhAa3. Values are means from a triplicate experiment. Error bars indicate ± SD. The significant fold changes (*P < 0.05 ± SD) between different *A*. *actinomycetemcomitans* strains were calculated by One-way ANOVA test with Tukey-Kramer test (**B**).

The *orf*X is a gene that is located immediately upstream of *ltx*CABD operon. It is co-transcribed with the *ltx* operon^4, 10, 14^. In the case of the hyper-producer with the 530 bp deletion, a portion of the gene *orf*X was deleted leading to transcriptional fusion between *orf*X and *ltx* operon (Fig. 2A). Further analysis of the *ltx* promoter deletion constructs for transcriptional fusion were carried out by RT-PCR using primers orfJnF and ltxCqR. The strains RhAa-*ltx*P298-530, RhAa-*ltx*P398-530, RhAa-*ltx*P451-530 and RhAa-*ltx*P298-397 in which *orf*X is present were compared with RhAa-*ltx*P530. RT-PCR results indicated that strains RhAa-*ltx*P298-530, RhAa-*ltx*P398-530, RhAa-*ltx*P451-530 and RhAa-*ltx*P298-397 did not have transcriptional fusion between *orf*X and *ltx* operon as indicated by a lack of amplification. RhAa-*ltx*P530 showed amplification of an intercistronic region indicating a transcriptional fusion between *orf*X and *ltx* operon (Fig. 3A). In addition, it was also shown that RhAa-*ltx*P530 with transcriptional fusion had significantly increased *ltx*A expression (*P* = 0.0002) compared to RhAa3 as evidenced by qRT-PCR. Nevertheless, the strains in which transcriptional fusion had not occurred, RhAa-*ltx*P298-397 (*P* = 0.0082) and RhAa-*ltx*P298-530 (*P* = 0.0449) had a significantly higher *ltx*A expression compared to RhAa3 (Fig. 3B). These results suggested that the 298–397 region could contain an element that can alter *ltx*A expression in RhAa3 strain. We have also analyzed whether successive deletions lead to in-frame fusion of *orf*X to *ltx*C by translating the nucleotide sequence (Data not shown). The results showed that none of the mutant strains had in-frame fusions due to deletion.

### Analysis of the leukotoxin promoter region 298–397 for NagC binding site

**Figure 4:**
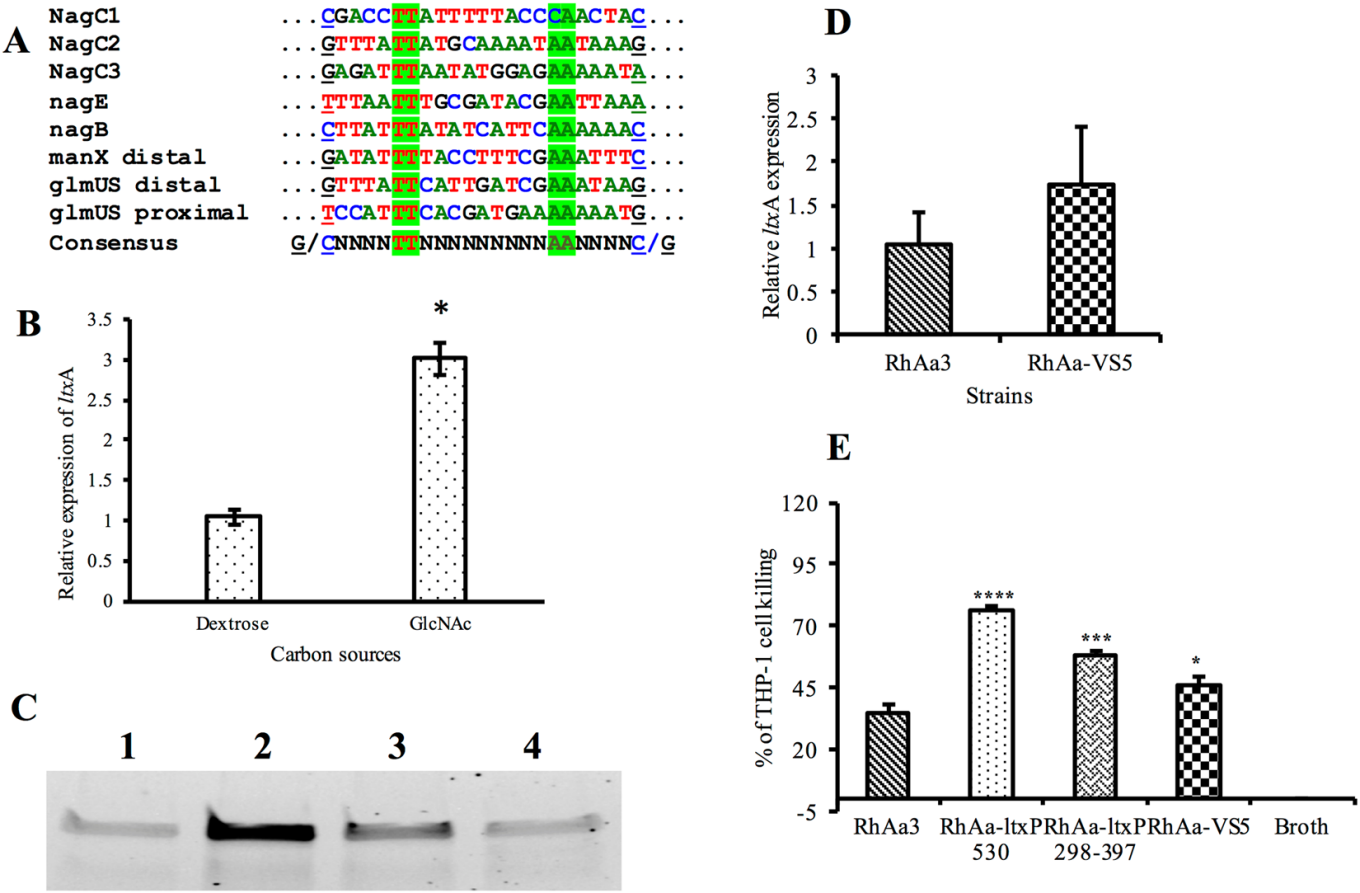
Prediction of NagC in leukotoxin regulation. *In silico* prediction of the NagC consensus binding site within the 530 bp of *ltx* operon promoter region. NagC site 2 and NagC site 3 are the predicted sites in the leukotoxin promoter region (See Supplement Figure S1). NagC site 2 is present within the region 298–397 in the 530 bp region (**A**). The relative *ltx*A expression upon supplementation of GlcNAc by RhAa3 was assessed by qRT-PCR in RhAa3 strain. Values are the mean of three independent replicate experiments. Significant difference between the group was calculated by Student’s *t* test *P* > 0.05 was considered as significance (**B**). Western blot analysis of the mutant strains showing the signals reacting to LtxA antibody; 1. RhAa3, 2. RhAa-*ltx*P530, 3. RhAa-*ltx*P298-397 and 4. RhAa-VS5 (**C**). qPCR analysis showed that there was no significant increase in *ltx*A expression in RhAa-VS5 compared to RhAa3 (**D**). The significant killing of THP-1 cells (*P* < 0.05) between RhAa3 and the strains were calculated by One-way ANOVA test. *indicates *P* < 0.001 ± SD with Tukey-Kramer test (**E**).

Based on previous studies and *in silico* analysis, we predicted a NagC (a transcriptional regulator) binding consensus sequence within the *ltx* promoter region 298–397 (Fig. 4A)^20^. Further analysis of the whole genome sequence database of *A*. *actinomycetemcomitans* (strain D7S NCBI accession number CP003496) showed that the homologous genes responsible for the metabolism of *N*-acetyl glucosamine (GlcNAc) are *nag*A: D7S_0040 and *nag*B: D7S_00401. To show that NagC is a negative regulator and that GlcNAc can repress *ltx*A expression in RhAa3 strain, the cells were grown in media containing either dextrose or GlcNAc. GlcNAc is the repressor for NagC and the binding of NagC to the *ltx*A promoter should be blocked in the presence of GlcNAc. Thus it was expected that *ltx*A expression would be increased in the presence of GlcNAc. It was interesting to note that *ltx*A expression by RhAa3 strain in the presence of GlcNAc was significantly increased (*P* = 0.001) when compared to the cells grown in dextrose containing media (Fig. 4B). These results suggest that NagC might be involved in *ltx*A expression by interaction in the promoter region between 298–397. To confirm the above result, qRT-PCR analysis and SDS-PAGE analysis were carried out in *nag*C-disrupted strain RhAa-VS5. Western blot analysis did not show increased leukotoxin production in RhAa-VS5 strain as compared to RhAa3 (Fig. 4C). Further analysis of *ltx*A expression by qRT-PCR also showed that there was no statistical difference (*P* = 0.216) between RhAa-VS5 and RhAa3 (Fig. 4D). To further evaluate the influence of the 298–397 region on leukotoxin production, western blot was carried out in comparison with strains RhAa3 and RhAa-*ltx*P530. It was observed that there was an increased leukotoxin production as demonstrated by THP-1 cell killing assay in RhAa-*ltx*P530 and RhAa-*ltx*P298-397 whereas a decreased expression was observed in RhAa3 (Fig. 4E).

### Presence of transcriptional terminator in the 298–397 region

**Figure 5:**
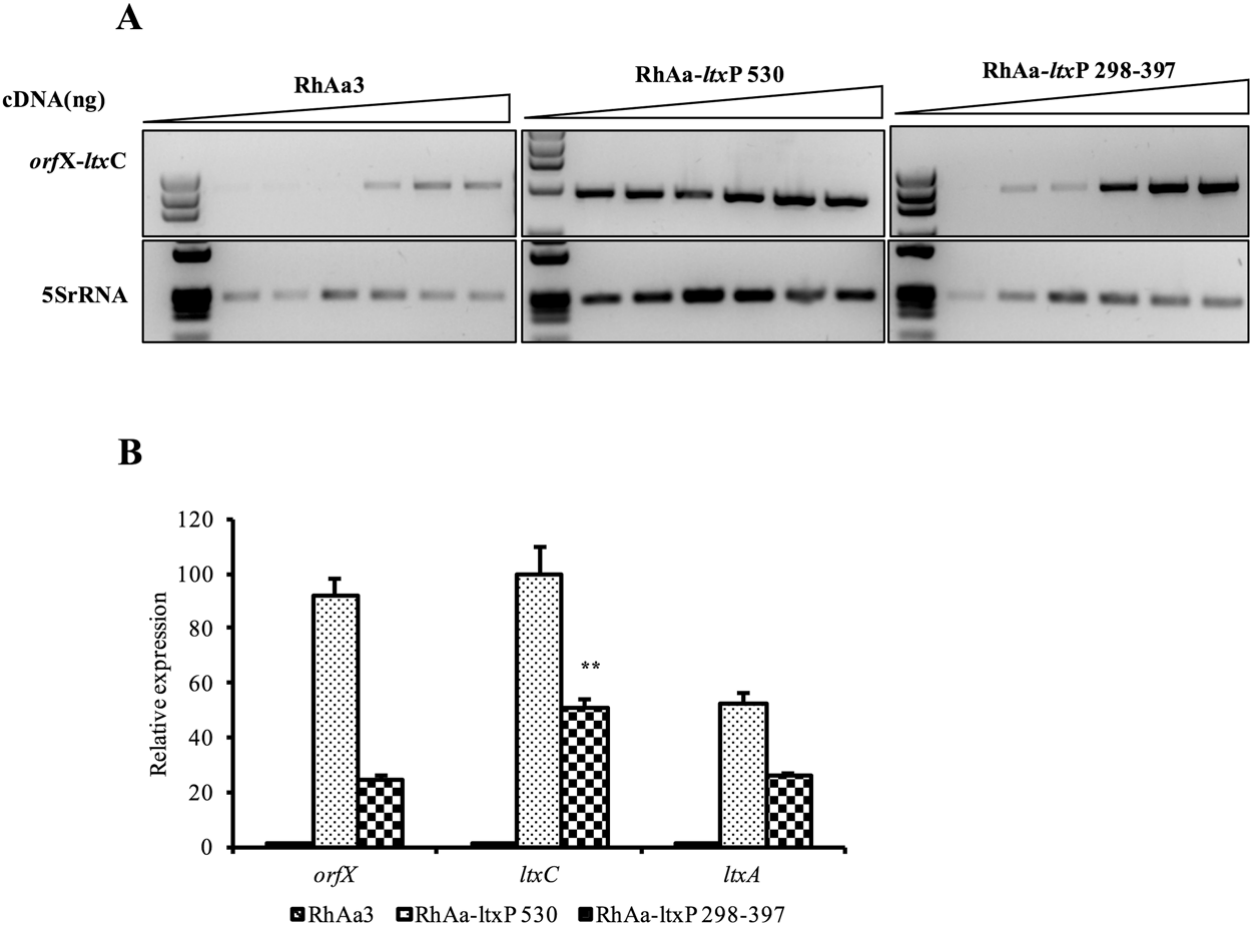
Assessment of a transcriptional terminator is present in the region 298–397. RT-PCR amplification of inter-cistronic region between *orf*X and *ltx*C in RhAa3, RhAa-*ltx*P530 and RhAa-*ltx*P298-397 with increasing concentrations of cDNA are shown as follows. Lane 1. 1 ng; Lane 2. 2 ng; Lane 3. 4 ng; Lane. 4 5 ng; Lane. 5 7.5 ng and Lane. 6 10 ng. Note that a clear signal was obtained from the 5 ng template in RhAa-*ltx*P298-397 (**A**). qRT-PCR shows significant increase in *ltx*C expression as compared to *orf*X expression in RhAa-*ltx*P298-397 further indicating a potential weak terminator, a negative regulatory element, within the region 298–397. Results are means ± SD for triplicate cultures normalized to 5S rRNA. The significant fold change was calculated by One-way ANOVA test. * indicates *P* < 0.001 ± SD with Tukey’s multiple comparison post-hoc test (**B**).

Semi-quantitative RT-PCR was carried out using the primers orfJnF-ltxCqR to show the presence of a weak terminator in the 298–397 region that could possibly decrease the transcription in the RhAa3 strain as compared to RhAa-*ltx*P530 and RhAa-*ltx*P298-397 (Fig. 5A). This result also implies that *orf*X is co-transcribed in the *ltx* operon as it is seen that with increasing cDNA concentrations. Amplification of the intervening region between *orf*X and *ltx*C occurred even in RhAa3. In addition, qRT-PCR analysis was performed to assess and compare the expression levels of *orf*X with *ltx*C and *ltx*A in the three strains, RhAa3, RhAa-*ltx*P530 and RhAa-*ltx*P298-397. Results indicated that the expression levels of *ltx*C was significantly increased in RhAa-*ltx*P298-397 (*P* = 0.0053) when compared to expression levels of *orf*X further indicating the presence of a terminator in the 298–397 region which slows down the transcription in the RhAa3 strain (Fig. 5B).

### Assessment of terminator strength

**Figure 6:**
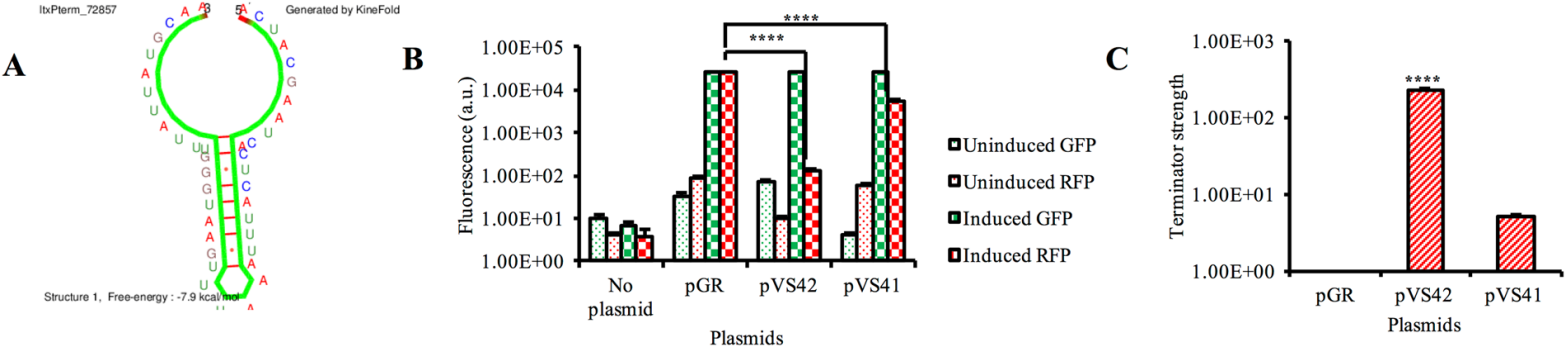
Transcriptional terminator in *ltx* promoter region. Putative terminator structure was predicted using KineFold software in the 298–530 bp region (**A**). The sequences were cloned in between GFP and RFP in a reporter plasmid, pGR. The expression of GFP and RFP fluorescence were measured before and after induction with arabinose. pGR without any cloned terminator sequence served as the negative control. The significant fluorescence (*P* < 0.05) between the samples was calculated by One-way ANOVA test, * indicates *P* < 0.001 ± SD with Tukey’s multiple comparison post-hoc test (**B**). The different terminator strengths were measured as described in results section. There was a presence of week terminator demonstrated in pVS41 (**C**).

*In silico* computational analysis of 298–397 region showed the presence of rho independent terminator loop structure with ΔG = −7.9 kcal/mol (Fig. 6A). Terminator strength (T_S_) was assessed as described previously^21^. The assay compared the expression of two fluorescent reporters, green fluorescent protein (GFP) and red fluorescent protein (RFP). The fluorescence data of the plasmid with no terminator, *rrnB* sequence (used as positive control) and sequences of interest are represented in Fig. 6B. Based on the T_S_ calculation, we found that *rrnB* is a strong terminator with T_S_ of 230.4 ± 21.1 and the 286 bp was found to have a weak terminator with a T_S_ of 5.3 ± 0.43 (Fig. 6C). However, it is not clear if the region has a Rho-independent or a Rho-dependent terminator.

### Mlc is an activator for *ltx*A expression in RhAa3

**Figure 7:**
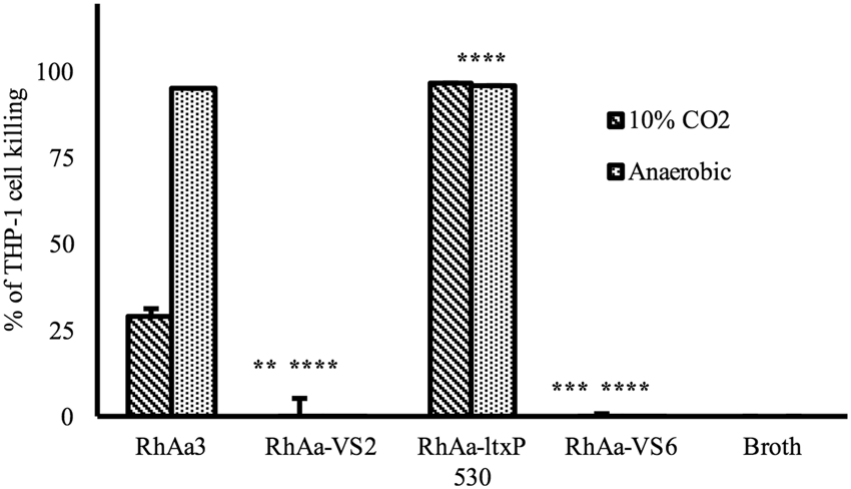
Leukotoxic activity of *mlc* disrupted strain. The comparison of leukotoxin production by RhAa3, RhAa-*ltx*P530 and RhAa-VS6 were evaluated using THP-1 human macrophage cell killing assay. Filter-sterilized culture supernatant from 24 h biofilm of RhAa3 showed significantly better killing than extract from RhAa-VS6. Uncultured growth media was used as negative control. The significant cell killing was calculated by One-way ANOVA test between different *A*. *actinomycetemcomitans* strains. Values indicate the mean percentage of THP-1 cell killing in a triplicate experiment. Error bars indicate SD. * indicates *P* < 0.001 ± SD with Tukey’s multiple comparaison test.

It has been previously shown that the Mlc binds upstream to the leukotoxin promoter and activates *ltx*A transcription. These results were described in a JP2 strain and it was shown that the *mlc* mutant resulted in decreased *ltx*A expression both under aerobic and anaerobic growth conditions. To test that a similar mechanism in RhAa3 strain, we created a *mlc* disruption strain RhAa-VS6 from RhAa3 strain and compared the leukotoxin production. We found that leukotoxin activity was significantly reduced in RhAa-VS6 strain under both 10% CO_2_ (*P* = 0.0004) and anaerobic conditions (*P* < 0.0001) (Fig. 7) indicating that the Mlc positively regulate *ltx*A expression.

## Discussion

The main aim of our ongoing studies was to assess the role of different *A*. *actinomycetemcomitans* virulence factors following implantation of these strains into the mouths of Rh monkeys. To this end, we have recently reported that a *lux*S deficient strain had a minimal impact on *A*. *actinomycetemcomitans* colonization while a leukotoxin null producer did not colonize supragingivally in the oral cavities of Rh monkeys^19^ (Unpublished data). The role of leukotoxin during periodontal disease progression and its contribution to the subgingival environment appears to be critical with respect to *A*. *actinomycetemcomitans* survival^15, 22–25^. After creating a leukotoxin knock-out strain our next goal was to create a hyper-producing *A*. *actinomycetemcomitans* strain by deleting the 530 bp promoter region for both *in vitro* and *in vivo* testing. Once accomplished, we wished to identify the specific region(s) within the 530 bp responsible for high levels of leukotoxin production. As a result, we created several strains that expressed differing levels of *ltx*A to assess regions critical for leukotoxin production.

A great deal of effort has been expended in attempts to understand increased leukotoxin production by using the JP2 strain of *A*. *actinomycetemcomitans*. This strain and those with similarly high levels of LtxA production have shown a naturally occurring deletion of 530 bp in its promoter region^15^. However, to date, no one has created a 530 bp deletion mutant and sequential deletions for comparison to a parental strain containing the wild type promoter. Our initial results showed that deletion of the 530 bp region led to leukotoxin production similar to that seen in the JP2 strains. As such, strains RhAa-*ltx*P98-530, RhAa-*ltx*P198-530, RhAa-*ltx*P298-350 and RhAa-*ltx*P298-397 all produced significantly increased levels of leukotoxin activity as compared to their wild type parental stain, RhAa3.

**Figure 8:**
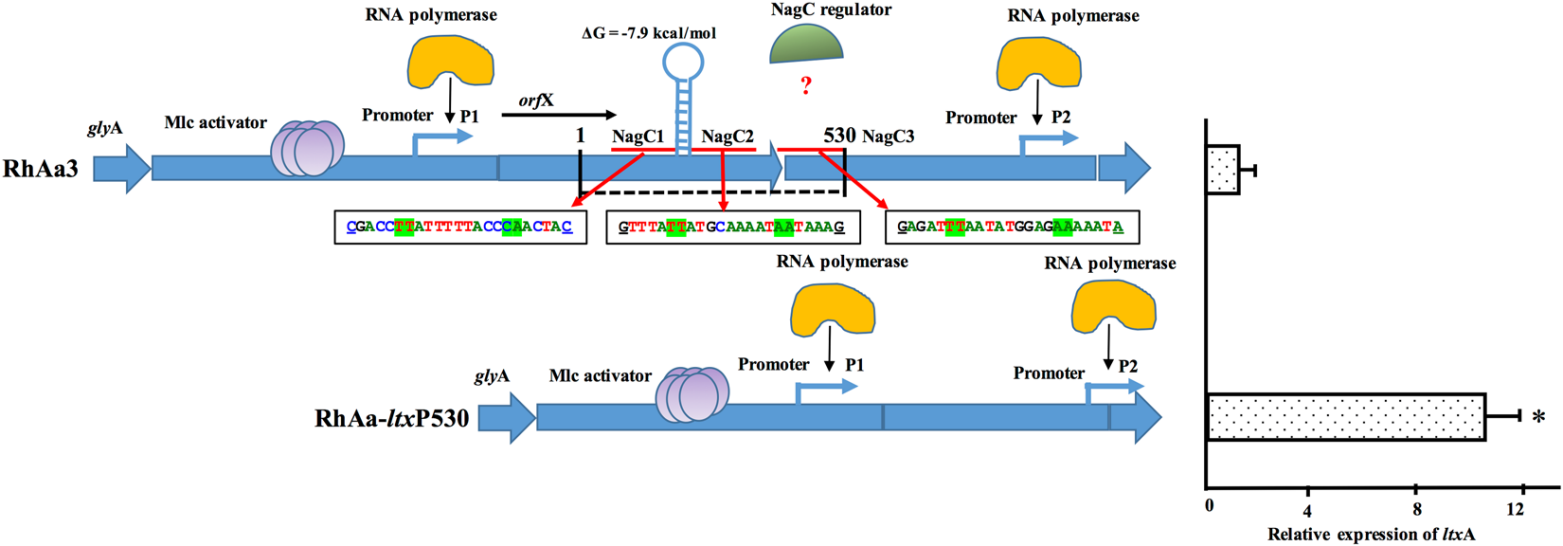
Schematic diagram showing the proposed model of transcriptional regulation of *ltx*A expression in RhAa3 and in RhAa-*ltx*P530 strains. The Mlc binding site is located preceding to *orf*X and the transcription of *ltx* operon appears to be under the direct influence of Mlc in the RhAa-ltxP530 strain. The expression level of Ltx is higher, which is shown in right side panel by graphical representation. In the RhAa3 strain due to the presence of a stem loop structure the transcription is reduced. There are three possible NagC binding sites which are predicted and shown in the red line. The sequences are shown below the RhAa3 panel. The green highlighted sequence shows TT or AA consensus bases. In the RhAa-*ltx*P530 strain the P1 promoter came closer due to the deletion and is one of the other mechanisms proposed for the high level of *ltx*A expression. *indicates *P* < 0.001 ± SD calculated by Student’s *t* test.

In order to understand the increased expression seen in RhAa-*ltx*P298-397 and possible regulatory mechanisms within that region that might be involved in leukotoxin production, region 298–530 was analyzed. It is known that an *orf*X gene is present upstream of *ltx*C in the 652 type promoter^15^ (Fig. 8). Examination showed that RhAa-*ltx*P298-530 and RhAa-*ltx*P298-397 deletion mutants demonstrated increased *ltx*A expression without transcriptional fusion of *orf*X. These results demonstrated the presence of regulatory elements within the 298–397 region. Initial RT-PCR experiments showed that transcriptional fusion between *orf*X and *ltx* operon was observed in RhAa-*ltx*P530, but not in RhAa3 and RhAa-*ltx*P298-397. However, when the same experiment was carried out with increasing concentrations of cDNA, the results showed that *orf*X was co-transcribed with the *ltx* operon in RhAa3 as previously reported in the JP2 strain^4, 10, 14^. The level of mRNA typically coincides with increased concentrations of cDNA. As seen in Fig. 5A, it is clear that the RT-PCR product appears in 2 ng of cDNA in RhAa-ltxP298-397 strain but is absent in the RT-PCR product of the WT RhAa3 strain. These results suggest that a repressor is found in the region 298–397 that causes decreased expression of *ltx*A in the wild-type strain. One of the limitations of this study is that occasionally we found there was no correlation found between the leukotoxicity and gene expressions analysis between the mutant strains.

At one point in time it was thought that there were two promoters, P_1_ and P_2_, in the JP2 strains that controlled *ltx*A expression^4^. Later, it was shown that only one promoter P_1_ was present and that there was no second initiation site for leukotoxin near *ltx*C in JP2 as well as a Y4 strain^10^. Hence, it was proposed that the regulatory elements controlling P_1_ in the JP2 strain may modulate *ltx*A expression in the 652 type promoter as well.

It has been shown that CRP and Mlc are regulatory proteins that activate leukotoxin expression in the JP2 strain^8^. In our experiments deletion of *mlc* also resulted in decreased leukotoxin expression. Further, the THP-1 cell killing assay showed significantly reduced killing by RhAa-VS6 strain (the *nag*C deletion mutant). These results suggested that the Mlc did regulate leukotoxin expression in the monkey derived WT strain RhAa3 confirming previous results^11^. We also suggest that the Mlc activates *ltxA* expression in the RhAa3 strain with a similar mechanism as seen in the JP2-like strains. The operator region for Mlc appears to be similar in both these strains. Furthermore, this is the first time this has been reported in a wild-type strain.

Based on the *in silico* prediction results, NagC was identified as another possible modulator of *ltx*A expression (Fig. 4A). NagC is a dual-function, activator-repressor, which acts by binding to two operators forming a DNA loop^26^. GlcNAc-6-P, the product of GlcNAc transport by PTS system is the specific inducer of NagC. Further, NagC and Mlc have almost identical amino acid sequences in their helix-turn-helix (H-T-H), DNA binding motif. In addition, the consensus binding sites are also very similar^20^. In agreement with previous results we also conclude that the Mlc protein acts as an activator for the expression of *ltx*A^11^. *In silico* computational analysis suggested the presence of a binding site for the NagC, a potential transcriptional modulator. Analysis of the *nag*C mutant RhAa-VS5, revealed that no significant changes were found in *ltx*A expression or production as evidenced by RT-PCR and western blot. A simple mechanism of gene regulation is seen in the competition between repressors and activators for common or overlapping DNA binding sites at the promoter regions^27^. In this case, it is possible that the condition we tested may favor binding of the activator, Mlc in its’ binding at, the position 298–397, while neglecting binding of NagC. It is also possible that the increased expression of *ltx*A suggests that NagC may have a role in activation in an indirect manner within the three predicted binding sites as demonstrated in Fig. 8. Therefore, at this point we speculate that NagC is not directly involved in *ltx*A expression. It is also possible that NagC can trigger other factors to act on *ltx*A expression. Further study is required to trace the protein-protein or protein DNA interactions that could lead to the over expression of *ltx*A.

Next, the region between 245–530 in the 530 bp region was analyzed for other regulatory elements. This region represents the intervening area between 3′ end of *orf*X and the end of the 530 bp deletion. The presence of a transcriptional terminator within the region was shown by *in silico* analysis using KineFold software (Supplementary Fig. S1). It was also shown that a stem-loop structure was found with a ΔG = −7.9 kcal/mol in the 298–397 region. The expression levels of *orf*X and *ltx*C were assessed for RhAa3, RhAa-*ltx*P530 and RhAa-*ltx*P298-397 by qRT-PCR. These results showed an increased expression of *ltx*C in RhAa-*ltx*P298-397 suggesting a negative regulatory element between *orf*X and *ltx*C. Our results suggest a potentially weak terminator, in this region. Terminator strength assessment was studied within this region. It was shown that a weak terminator was present within the 298–530 bp region.

It is known that transcription terminators can be present at the end of genes or in the upstream regulatory regions^28^. Terminators at the end of the genes prevent unintended transcription in downstream genes^29^. Terminators in the regulatory regions are known to regulate the expression of the structural gene in response to mechanical and environmental stimuli^29^. Based on these findings, we speculate that there is a potential negative regulator, which acts as a weak terminator, in the region between 298–530 in the 530 bp region. The terminator could be a Rho-dependent or a Rho-independent terminator^30, 31^. It is highly likely that multiple factors control leukotoxin expression.

In conclusion, we confirmed the fact that deletion of 530 bp in the structural leukotoxin promoter region increased *ltx*A expression. We also confirm that the structure of this regulatory region within the promoter of RhAa-*ltx*P530 and RhAa3 strains are alike and can be regulated in a different manner. Further, in our study deletion of fewer than 530 bp (deletion of 298–397 bases) resulted in increased leukotoxin expression. These results suggest the presence of regulatory elements within specific sites in the 530 bp region control *ltx*A expression. Analysis of terminators and terminator strength showed the presence of a possible weak terminator in the region between 298–530 that can influence *ltx*A expression. Moreover, our results confirmed the observation that Mlc controls leukotoxin expression in our wild type RhAa3 strain as has been shown in the JP2 strain^11^. Further analysis of the exact region of the terminator and other possible regulatory elements in the leukotoxin promoter region is required. This newly developed strain will make it possible to study various strains of *A*. *actinomycetemcomitans* that differentially expresses *ltx*A *in vivo* in the oral cavity of monkeys.

## Methods

### Bacterial strains, growth conditions and plasmids

The strains and plasmids used in this study are listed in Table 1. A minimally leukotoxic 652 type *A*. *actinomycetemcomitans* (RhAa3) was used in this study^19, 32^. *A*. *actinomycetemcomitans* strains were routinely grown on Brain Heart Infusion (BHI) agar (BD company, NJ) supplemented with 0.6% yeast extract, 0.8% dextrose and 0.4% sodium bicarbonate. When necessary 0.8% dextrose was replaced with *N*-Acetyl-D-glucosamine (GlcNAc) (Sigma, St. Louis, MO). *A*. *actinomycetemcomitans* strains were incubated at 37 °C in a 10% CO_2_ or in a, anaerobic incubator for 16–48 h. *Escherichia coli* strains were routinely grown on LB broth or LB agar media supplemented with appropriate antibiotics in a 37 °C aerobic chamber.

**Table 1:** Plasmids and bacterial strains used in this study.

Plasmid/bacterial strain	Relevant genotype/characteristics	Source
*E*. *coli* Stellar™ Competent Cells	*F–*, *endA1*, *supE44*, *thi-1*, *recA1*, *relA1*, *gyrA96*, *phoA*, *Φ80d lacZΔ M15*, *Δ* (*lacZYA - argF*) *U169*, *Δ* (*mrr - hsdRMS - mcrBC*), *ΔmcrA*, *λ−*	Clone tech laboratories Inc, CA
RhAa3	Wild type strain	19
JP2	A hyper-leukotoxic strain	40
RhAa-VS2	Sp^R^ *ltx*A disrupted strain	19
RhAa-VS5	*nag*C disrupted RhAa3	This study
RhAa-VS6	*mlc* disrupted RhAa3	This study
RhAa-*ltx*P530	530 bp of *ltx*A promoter deleted strain	This study
RhAa-*ltx*P98-530	432 bp of *ltx*A promoter deleted strain	This study
RhAa-*ltx*P198-530	332 bp of *ltx*A promoter deleted strain	This study
RhAa-*ltx*P298-530	232 bp of *ltx*A promoter deleted strain	This study
RhAa-*ltx*P398-530	132 bp of *ltx*A promoter deleted strain	This study
RhAa-*ltx*P451-530	79 bp of *ltx*A promoter deleted strain	This study
RhAa-*ltx*P298-397	99 bp of *ltx*A promoter deleted strain	This study
pJT1	Sp^R^; gene disruption plasmid for *A*. *actinomyctemcomitans*	33
pGR	Reporter plasmid for measuring terminator strength	21
pVS31	Deletion plasmid to create RhAa-*ltx*P530 strain	This study
pVS32	Deletion plasmid to create RhAa-*ltx*P98-530 strain	This study
pVS33	Deletion plasmid to create RhAa-*ltx*P198-530 strain	This study
pVS34	Deletion plasmid to create RhAa-*ltx*P298-530 strain	This study
pVS35	Deletion plasmid to create RhAa-*ltx*P398-530 strain	This study
pVS36	Deletion plasmid to create RhAa-*ltx*P451-530 strain	This study
pVS37	Deletion plasmid to create RhAa-*ltx*P298-397 strain	This study
pVS41	Promoter regions sequence from 298–530 cloned between gfp and rfp of pGR *rrn*B	This study
pVS42	Terminator cloned between *gfp* and *rfp* of pGR	This study

### Biofilm growth conditions

*A*. *actinomycetemcomitans* strains from −80 °C freezer were streaked on to brain heart infusion agar (BHI) plates containing yeast extract (0.6%), sodium bicarbonate (0.4%), dextrose (0.75%) and 1.5% agar. After 24 hr growth in a 37 °C incubator at 10% CO_2_/90% air atmosphere, a single colony was picked from the well separated area of the agar plates and suspended in BHI broth (BHI) containing yeast extract (0.6%), sodium bicarbonate (0.4%) and glucose (0.75%). Single colony was picked from the well-separated area from the agar plates, suspended in BHI broth containing yeast extract (0.6%), sodium bicarbonate (0.4%) and glucose (0.75%). The aggregated cells from the colony were disrupted using a hand held homogenizer (Kimble Chase, Vineland, NJ) and the non-aggregated free cells were removed by leaving the suspension on ice for two minutes. The cell density of the top portion was adjusted to ~10^8^ cells per ml (OD_600_ = 0.7–0.8). These cells were used as the inoculum for the bio film growth. Biofilms were grown on brain BHI broth for 24 hr in a 37 °C incubator at 10% CO_2_/90% air atmosphere or in a 37 °C anaerobic chamber containing 80% N_2_, 10% CO_2_ and 10%H_2_. For testing the leukotoxic activity of each strain by THP-1 cell killing assay and western blot, 2 ml of supernatant was collected and passed through a 0.2 μ filter and used immediately or stored at −80 °C freezer. The mature biofilm cells were washed with PBS, cells were collected using a cell scrapper and stored in RNA stabilizing solution (ice cold 0.9% saline supplemented with 1/10^th^ volume of 95% ethanol and 5% citric acid saturated phenol mixture).

### DNA manipulations

PCR amplifications were performed using Phusion DNA polymerase (Thermoscientific, Waltham, MA). Oligonucleotides used in this study were synthesized from Integrated DNA technologies (Coralville, IA) and are listed in Supplementary Table 1. Genomic DNA plasmid and gel extraction kits were purchased from Qiagen (Qiagen, Foster City CA) and used as per manufacturer’s instruction. Restriction enzymes were purchased from New England Biolabs (New England Biolabs, Inc. Ipswich, MA) and used as per the manufacturer’s directions. Stellar competent cells were transformed as instructed in manufacturer’s manual (Clontech^®^, Mountain View, CA). The gene disruption and promoter deletion plasmids were electroporated as described previously^19^. All plasmid constructs and mutant *A*. *actinomycetemcomitans* strains were verified by DNA sequencing (Macrogen Inc, New York, NY).

### Construction of *ltx*A promoter deletion in *A. actinomycetemcomitans*

A series of deletions within the *ltx*A promoter was carried out in RhAa3 as described previously^33^. Initially, a JP2 like 530 bp *ltx*A promoter strain was created. The aim was to mimic the JP2-type promoter by deleting the 530 bp in the promoter region. A scarless, marker-less deletion approach was used to construct a hyper-LtxA producing strain as described previously^33^. Primers 530USSUpF, 530UpR and 530DnF, 530DnR were used to amplify the upstream and downstream flanking regions of the 530 bp region in the *ltx*A promoter region to be deleted. Both the fragments were amplified with 15 bp complementary to each other to enable fusion between them. In addition, restriction sites *Xma*I and *Sac*I were introduced into the 5′ and 3′ ends of the flanking fragments respectively by PCR to enable directional cloning into pJT1. Overlap extension PCR was performed as described previously^34^ with the first PCR step using equimolar concentrations of the upstream and downstream flanking fragments. The end primers were added and the second PCR step using 5 µl template from the first PCR step. The fused PCR amplified fragment was then digested with *Xma*I and *Sac*I and ligated into restriction digested pJT1. The resultant plasmid was designated pVS30. The plasmid pVS30 was electroporated into RhAa3 and the transformants were screened by PCR using primers 530bpscreenF and 530bpscreenR. The resultant strain was designated as RhAa-*ltx*P530.

The successive *ltx*A promoter deletion mutants were created as follows. PCR products containing different lengths of the leukotoxin promoter along with portions of upstream gene *gly*A and downstream *ltx* operon were amplified using primer sets 530USSUpF, 400UpR and 400DnF, 530DnR; 530USSUpF, 300UpR and 300DnF, 530DnR; 530USSUpF, 200UpR and 200DnF; 530DnR; 530USSUpF, 100UpR and 100DnF, 530DnR; 530USSUpF, 80UpR and 530DnF, 80DnR; 530USSUpF (Supplementary Table S1). The upstream and downstream amplified products from each set were fused by overlap extension PCR as described above. The fused fragments were digested with *Xma*I and *Sac*I and ligated into pJT1. The resultant plasmids were designated pVS31-pVS35. The plasmids were transformed into RhAa3 by electroporation as described previously and the strains were designated as RhAa-*ltx*P98-530, RhAa-*ltx*P198-530, RhAa-*ltx*P298-530, RhAa-*ltx*P398-530 and RhAa-*ltx*P451-530 respectively (Table 1). Another leukotoxin promoter deletion was made in a similar way with minor modifications. The primer sets used for amplification of upstream and downstream fragments were 530UpFI, -nagUpR and -nagDnF, 530DnRI. Fusion of the fragments into the vector was done by In-Fusion HD cloning (Clontech^®^, Mountain View, CA) as per the manufacturer’s instructions. The vector pJT1 was linearized with *Xho*I and *Not*I. The gel purified fragments and the linearized vector were incubated with the 5X In-Fusion HD Enzyme premix at 37 °C for 15 min followed by 50 °C for 15 min. The mixture was then transformed into Stellar competent *E*. *coli*. The resultant plasmid was designated as pVS36 after confirmation by sequencing. The plasmid was electroporated into RhAa3 as previously described and the mutant was designated as RhAa-*ltx*P298-397.

### Construction of *nag*C and *mlc* mutants

Scarless markerless deletion of *nag*C and *mlc* were carried out in RhAa3 strain as described previously^33^. Briefly, the flanking regions of *nag*C (Acc. No. CP003496 locus id. D7S_00428), were amplified with nagCDNFNew, NAGC3R and 3619 R, nagCURnew primer. In the case of *mlc* (Acc. No. CP003496 locus id. D7S_02207), the flanking regions were amplified using primer sets mlcUF, mlcUR and mlcDF, mlcDR. The PCR amplified flanking fragments were cloned at *Xho*I and *Not*I sites of pJT1 plasmid using In-Fusion HD cloning kit as per the manufacturer’s instructions. The resultant *nag*C and *mlc* knockout plasmids were designated as pVS39 and pVS40 respectively. The plasmids pVS39 and pVS40 were electroporated into RhAa3 and the *nag*C and *mlc* mutants were screened with primer sets NAGC4R, 3843 R and mlc5′, mlc3′ respectively. The *nag*C mutant was designated as RhAa-VS5 and *mlc* mutant was designated as RhAa-VS6.

### THP-1 cell killing assay

The leukotoxic activity of different *A*. *actinomycetemcomitans* strains were assessed by THP-1 cell killing assay as described previously^35^. Cell viability was the measure of luminescence using the microplate reader (Infinite M200pro, Tecan, Austria GmbH, Austria) which was proportional to the amount of ATP released by the live cells. In all experiments, uncultured liquid media and purified leukotoxin were used as negative and positive controls respectively.

### SDS-PAGE and western blot analysis of LtxA

Protein samples were processed and separated onto SDS-PAGE gel (Bio-Rad, Hercules, CA), stained with SYPRO^®^ Ruby and the image was captured as described in the product manual (Thermoscientific, Waltham, MA). SeeBlue^®^ Plus2 Pre-Stained standard was used to determine the protein molecular weight (Invitrogen, Carlsbad, CA). Protein samples were subjected to western blot analysis as previously described^36^. Briefly, protein bands were transferred from SDS-PAGE onto a nitrocellulose membrane, probed with polyclonal anti-LtxA antibody (1:2000-Monoclonal anti-mouse antibody, ProMab, Richmond, CA) and peroxidase labeled goat-anti-mouse antibody (1:10,000, Sigma, St. Louis, MO). Reactive bands were visualized by treating the membrane with Super signal West Femto substrate (Pierce, Rockford, IL) and exposing the membrane to FluorChem Q system (San Jose, CA).

### RNA extraction from biofilm, RT-PCR and qRT-PCR analysis

RNA was isolated as previously described^19^. The RNA was purified using Micro Bio-Spin P-30 Gel Columns (Bio-Rad, Hercules, CA) and treated with DNaseI and RNA purification kit (Zymo Research, Irvine) to remove genomic DNA contamination. PCR using *ltx*A primers was used to confirm the elimination of genomic DNA contamination before proceeding to qPCR.

Transcriptional fusion analysis was performed to demonstrate whether the difference in leukotoxin expression was as a result of transcriptional fusion between *orf*X and *ltx*A operon in the created mutant strains as described previously^37^. To do this we carried out two step RT-PCR. The total RNA was extracted from the strains and 2 μg of total RNA was converted into cDNA in the first step as described in manufacturer’s instruction (Applied Biosystems, Foster City, CA). A second PCR step was carried out with primers OrfXJnF and ltxCqR using the cDNA template. The forward primer was designed to prime at the 5′ end of *orf*X gene and the reverse primer was used in the case of *ltx*C. The DNA and RNA were used as positive and negative controls in the second PCR step reactions respectively. To further analyze if there was a low level of transcript in the inter cistronic region, increasing concentrations of cDNA template was used for second RT-PCR step.

For qRT-PCR, LightCycler480 system was used using Roche SYBR green master mix. cDNA was used in the dilution of 1:100 for reference gene 5SrRNA and 1:25 was used for target genes as described previously^19^. The cycling condition was followed as described in the product manual (Roche Life Science, Indianapolis, IN). Melting curve analysis was done to assess product specificity. Data analysis was done using LightCycler 480 software (Version 1.2.9.11).

### Transcriptional terminator assessment

The stem loop structure in the RNA secondary structure was predicted using KineFold web server^38^. The plasmid pGR (A generous gift from Christopher Voigt-Addgene plasmid #46002) was used for terminator assessment assays. The terminator sequence of interest was amplified with primers pGR153F and pGR286R using the RhAa3 DNA as template. As a control, *rrnB* terminator was amplified from pJAK16 using primers pGRrrnBF and pGRrrnBR. All the sequences were cloned at *Eco*RI and *Spe*I sites of pGR plasmid using an In-Fusion HD cloning kit as described in the product manual. The resultant plasmids were designated as pVS41 and pVS42 respectively. The transcriptional terminator strength assays were performed in Stellar *E*. *coli* as described previously^21^. The cell growth and collection of cells were carried out as described previously^39^. LSRFortessa X-20 flow cytometer (BD Biosciences, CA) was used for flow cytometry measurements as described previously^21^. GFP data was extracted from FITC and RFP data from PE-CF594 channels respectively. GFP and RFP fluorescence was the measure of the geometric means of the data obtained from FITC and PE-CF594 channels respectively. The data was analyzed using FlowJo 9.9.4. T_S_ and was calculated by applying the equation; T_S_ = 1/1 − T_E_ = [(GFP)_*Term*_/(RFP)_*Term*_] [(GFP)_*0*_/(RFP)_*0*_]^−1^, where T_E_ refers to terminator efficiency, (GFP)_Term_ and (RFP)_Term_ is the averages of populations measured by flow cytometry when terminator is present and (GFP)_0_ and (RFP)_0_ refers to the measurements of the control plasmid with no terminator present. T_S_ = 1 refers to no termination. The data presented are as means ± SD. *P < 0.05.

### Statistical Analysis

In all cases where two samples were compared we used a Students-t-test to determine statistically significant differences using a level of *P* < 0.05 as our end point determinant. In any case where three of more comparisons were being assessed we used ANOVA and relied on Tukey-Kramer test to discriminate between means that were significantly different setting the level at *P* < 0.05.

## Electronic supplementary material

Supplementary file
