# Supplementary material for: Increased leukotoxin production: Characterization of 100 base pairs within the 530 base pair leukotoxin promoter region of Aggregatibacter actinomycetemcomitans
Source: Sci Rep. 2017 May 15;7:1887. doi: 10.1038/s41598-017-01692-6 (PMC5432517; doi:10.1038/s41598-017-01692-6)
Supplement: Supplementary file 1 — Supplementary file [file 41598_2017_1692_MOESM1_ESM.pdf]

Increased leukotoxin production: Characterization of 100 base pairs within the 530 base pair leukotoxin promoter region of *Aggregatibacter actinomycetemcomitans*

Vandana Sampathkumar [1+], Senthil Kumar Velusamy[1+], Dipti Godboley[1], Daniel H. Fine[1*]

[1] Department of Oral Biology, Rutgers School of Dental Medicine, Newark, NJ 07103 USA

[+] These authors contributed equally to this work

[*]Correspondence:

Daniel H. Fine

185 South Orange Avenue

Medical Science Building Rm C-636

Newark New Jersey, 07103

Phone: 973-972-3728

FAX: 973-972-0045

Email: finedh@sdm.rutgers.edu

Supplementary Table. S1. Oligonucleotides used in this study

Oligonucleotides	Sequence (5’→3’)
***ltx*A promoter deletion**	
530USSUpF	ATACTCCCGGGAAGTGCGGTGATTCTTTCTTCTTGCGGTGATGAAG
530UpR	CTAAATAAAATAACCAAACCACAATATCCAGTATGCCATCATTAAAAG
530DnF	CTTTTAATGATGGCATACTGGATATTGTGGTTTGGTTATTTTATTTAG
530DnR	TACTACGAGCTCGCAGGAGCAAATAAAGTTAAGCC
400UpR	CTAAATAAAATAACCAAACCACAATAGTTGGTAAAATTAGATTTAAAATG
400DnF	CATTTTAAATCTAATTTTACCAACTATTGTGGTTTGGTTATTTTATTTAG
300UpR	CTAAATAAAATAACCAAACCACAATAGTCTGCGTAATAAGCAAGAGAAG
300DnF	CTTCTCTTGCTTATTACGCAGACTATTGTGGTTTGGTTATTTTATTTAG
200UpR	CTAAATAAAATAACCAAACCACAATAATTTTTAAAATTACAATGGAAAC
200DnF	GTTTCCATTGTAATTTTAAAAATTATTGTGGTTTGGTTATTTTATTTAG
100UpR	CTAAATAAAATAACCAAACCACAATATATTTAAGCTTTATTATTTTGC
100DnF	GCAAAATAATAAAGCTTAAATATATTGTGGTTTGGTTATTTTATTTAG
80UpR	ATAAAATAACCAAACCACAATATTGTTAAACAACAAATAGAAATATAAC
80DnF	GTTATATTTCTATTTGTTGTTTAACAATATTGTGGTTTGGTTATTTTAT
530UpFI	GGGCCCAATTAATGGCGATTCTTTCTTCTTGCGGTGATGAAG
530DnRI	TACTAGTTCGAATAACGCAGGAGCAAATAAAGTTAAGCC
-nagUpR	CTTATTTAACCTTATATTATAGGAATAATTTTTAAAATTACAATGGAAAC
-nagDnF	GTTTCCATTGTAATTTTAAAAATTATTCCTATAATATAAGGTTAAATAAG
***nag*C disruption**	

NAGC3R	TACTAGTTCGAATAACAGGCGGATATTGTGATCAGTTCC
nagCDNFNew	CGCACTTTAAGGCTGTAAACGCTTTTTTCTCCCGAAAAGATAAG
3619R	GGGCCCAATTAATGGCGAATGGTGCGGGACGAGGGACTTG
nagCURNew	CTTATCTTTTCGGGAGAAAAAAGCGTTTACAGCCTTAAAGTGCG
***nag*C mutant screening**	
NAGC4R	GAGGTGTTGGTGGAAAAGCTGG
3843R	CCAATAAATTGTATAGTGGAAGC
mlc disruption	
mlcUF	GGGCCCAATTAATGGCCGTTCTTCACGTTGCGAACCGCC
mlcUR	ATTGAGCTAAAACAAAGTTTAAATAATTCCGTATTTTTTA
mlcDNF	TAAAAAATACGGAATTATTTAAACTTTGTTTTAGCTCAAT
mlcDNR	TACTAGTTCGAATAACCTATGCTTTATACCAAGCTCAC
mlc mutant screening	
mlc5’	CCGTGCCGTAACGACGCAAATCAC
mlc3’	GTCGGAGTCGGCATTTATTCGAC
qRT-PCR & RT-PCR	
orfXqF	CACTTTGTGAATACTGGCAACTT
orfXqR	GTGCCAATGATTTGTGCGAATA
orfXJnF	CGAGTATGTCCAGTACAGAATATGC
ltxAqF	GCGGCTCTATTACTTGCA
ltxAqR	GCAATACCTAGGAAAGAC
ltxCqF	GGGCAAATTTAAGCCTTGAG

ltxCqR	GCGCAATCCAATCAATAAAC

Sequences underlined are the restriction enzyme recognition sites

Supplementary Figure. S1. The nucleotide sequence showing 530 bp ltxA promoter deletion and regulatory elements binding sites

The *gly*A stop is shown as the stop codon for *gly*A gene. The Mlc binding site is highlighted in bold face. A -35 and -10 region preceding to *orf*X is highlighted. The start codon of *orf*X is ATG and is shown in bold face and the orfXJnF primer (in blue) binding site is highlighted. The 530 bp deletion starts from 1 to 530 and is shown in small letters. The *orf*X stop (tag) codon is shown in bold face. The encircled (red) bases are the proposed predicted stem loop structure. The probable NagC binding sites were also highlighted. The *ltx*C SD sequence and the start codon are highlighted in bold face.

```
 ←glyA stop
ltxP  TAATTTTAATCAAACGAAAAAAGCAAAGCGGCAATGAAAATTGCCGCTTTTTCTTTTTGAGAAATATGGTAGTCAAAATTTTGCAGACCAAAACCTGATA
                                                                                            Mlc binding
ltxP  AAAGTATTTTCTCAGTCTAATTTTTGCGTATTAATACAATACGAGATTGCGTTGGTAAAGTGTGATCAAAAAACTAATAATTTTATGAAATTAAATAATT
          -35                  -10
ltxP  TTTTCTATTGACTATTAAATAATCCGGAGTAAATTAGTCTCCAAAATTAACCAAAACTAGGTAATTTATCCGGTCAAAGGTTATCTTAAGTATTAACCCT
                    →orfX start orfXJnF                                                        orfXqF
ltxP  AAGGAAAAGGAAAACGAGTATGTCCGGTACAGAATATGCTCCATTTTATCTCCGTTTTATTCAGTTCCCAAATAATGAAGTTTCACTTTGTGAATACTGG
                                                         orfXqR
ltxP  CAACTTGTTCAGAATTCTGTAAAAAAGATCAGAAGAATAACGGTAATATTCGCACAAATCATTGGCACTTTCAGCGAAAAAGCTATTTGGAAATACCAAG
                         →530 bp deletion start
                         1         10        20        30        40        50        60        70
                         |....|....|....|....|....|....|....|....|....|....|....|....|....|....|....|
ltxP  GTACTTTTAATGATGGCATACTGGAagatgaagtagctaaagacgtttccccttactttaagaaatagtgccttacttgtcgcaagtgccatagttatcca
          80        90        100       110       120       130       140       150       160       170
      ....|....|....|....|....|....|....|....|....|....|....|....|....|....|....|....|....|....|....|....|
ltxP  ttttaaatctaattttaccaaccttctcatactgtcacagattacgcaatatcatagctattaccctgtactcaaaaaaagtaaatacttccctctctac
                                                         orfX stop←
          180       190       200       210       220       230       240       250       260       270
      ....|....|....|....|....|....|....|....|....|....|....|....|....|....|....|....|....|....|....|....|
ltxP  ttctcttgcttattacgcagacgattgactgaatttaaaatcacccttctaccgttgccatggggctagctgccatatagctgtgaagatcaaatcccgg
                                                   NagC 1
          280       290       300       310       320       330       340       350       360       370
      ....|....|....|....|....|....|....|....|....|....|....|....|....|....|....|....|....|....|....|....|
ltxP  tttccattgtaattttaaaaatatataagaaataatttgaagccgaccttattttacccaactacgaatcactcatttaaatttgaatgggtttattat
      NagC 2                                                   NagC 3
          380       390       400       410       420       430       440       450       460       470
      ....|....|....|....|....|....|....|....|....|....|....|....|....|....|....|....|....|....|....|....|
ltxP  gcaaaataataaagcttaaatatattcctataatataaggttaaataagttatatttTATTTGTTgTTAACAATaataattaaatcatagtctatttg
                                   530 bp deletion end←
          480       490       500       510       520       530   -10
      ....|....|....|....|....|....|....|....|....|....|....|....|....                        SD sequence
ltxP  atttcgtaatgagtgtggcatttttctgtcacgctatagtgtaagttatttgtgTATTGTGGTTTGGTTATTTTATTTAGAATAAATTATTAACAAGGA
          → ltxC start
ltxP  GATTTAATATGGAGAAAAATAATAATTTTGAAGTGTTAGGGTATGTGGCTTGGTTATGGGCAAATTCTCCTTTGCATCGAAATTGGTCTTTATCATTACT

ltxP  TGCTATTAATGTACTGCCTGCAATTCAATATGGGCAATATACATTATTAATGAGAGATGGGGTTCCTATTGCTTTTTGTA→ltxC,ltxB and ltxD
```
